# Developing a Research Agenda for HiAP Implementation: A Response to the Recent Commentaries

**DOI:** 10.34172/ijhpm.2023.8326

**Published:** 2023-11-26

**Authors:** Maria Guglielmin, Ketan Shankardass, Patricia O’Campo, Ahmed M. Bayoumi, Lauri Kokkinen, Carles Muntaner

**Affiliations:** ^1^Department of Health Sciences, Wilfrid Laurier University, Waterloo, ON, Canada; ^2^MAP Centre for Urban Health Solutions, Li Ka Shing Knowledge Institute, Toronto, ON, Canada; ^3^Dalla Lana School of Public Health, University of Toronto, Toronto, ON, Canada; ^4^Division of General Internal Medicine, St. Michael’s Hospital, Toronto, ON, Canada; ^5^Department of Medicine, University of Toronto, Toronto, ON, Canada; ^6^Institute of Health Policy, Management and Evaluation, University of Toronto, Toronto, ON, Canada; ^7^Faculty of Social Sciences, Tampere University, Tampere, Finland; ^8^Bloomberg School of Nursing University of Toronto, Toronto, ON, Canada

 The three commentaries by Williams and Valentine,^1^ Breton and Le Bodo,^2^ and Amri and Bump,^3^ written in response to our article,^4^ raise important points that highlight key gaps and challenges in studying and evaluating Health in All Policies (HiAP) initiatives. We appreciate and agree with many points raised about the complexity of HiAP initiatives; the importance of studying the role of non-governmental actors involved in local HiAP initiatives; and the need for greater focus on issues of health equity. In this correspondence, we outline how these ideas can inform a research agenda about the implementation of HiAP.

 Williams and Valentine^1^ aptly point out that HiAP is a complex intervention, describing it as “support(ing) actors and actor-groupings, who do not behave in predictable linear patterns, to work across organizational hierarchies, cultures, and disciplines to generate improved solutions.” Breton and Le Bodo^2^ also highlight that our analysis of HiAP implementation in Kuopio focused on the role of local state actors, which risks oversimplifying causal processes. Elsewhere,^5^ and in our current work to examine five case studies of HiAP implementation at the local level in Ontario and Québec,^6^ we include a focus on extra-governmental actors including from the private sector, third sector, and First Nations, as well as the influence of other government systems. Nevertheless, Breton and Le Bodo^2^ enrich the conversation on HiAP implementation by highlighting the essential role of dedicated coordination staff in some regions of France to facilitate contracts between Regional Health Agencies and local authorities to improve population health. While much of the scholarship on HiAP implementation focuses on political leaders and actors representing various policy sectors, this draws attention to the value of funding staff positions that make intersectoral coordination more feasible. This dovetails nicely with other recent research that expands on the role of “boundary spanners”^7^/“boundary spanning”^8^ in HiAP implementation, and more research in this area is certainly warranted to understand how to translate good intentions into action on health equity.

 We likewise agree with Breton and Le Bodo^2^ that systems theory can help understand generative causality through identifying and analyzing the system of actors and the connections they create that can lead to emergent properties that mobilize new agents and resources for HiAP implementation. While the case study of Kuopio did not centre on a systems theory approach, we have contributed to the development of a systems level approach to HiAP implementation,^5^ with a focus on understanding the relationships between key actors and resources across three sub-systems of government and a range of extra-governmental actors. Systems theory can help researchers and practitioners explain unexpected implementation outcomes. We have also argued that using systems theory can support researchers in studying complexity by informing hypotheses about the mechanisms of HiAP implementation and contributing to theory about how mechanisms across government sub-systems and other systems (including extra-government actors) are related.^5^

 It is also worth noting that the explanatory case study approach used to examine HiAP implementation in Kuopio is rooted in a realist ontology, which is congruent with a systems theory approach. Realism focuses research on identifying how specific intervention strategies trigger mechanisms that cause outcomes. It also acknowledges that outcomes are emergent from a broader context (that is part of a larger system).^5,9^ By developing and testing detailed hypotheses about the role of mechanisms, this approach contributes to middle range theory^10^ and a more complete picture of HiAP implementation – and relevant systems – over time. Our analysis of Kuopio addresses complexity by learning about HiAP implementation at an interpersonal level; whereas, a systems theory approach could extend the analysis by clarifying how and why contextual factors are interconnected.

 Williams and Valentine^1^ note that Finland’s unique context including its strong history^11^ of egalitarianism and global leadership in the HiAP approach^12^ may have contributed to greater ease in HiAP implementation, thus impeding transferability of findings. The realist approach that we adopt in our research assumes that *every *context is unique but that mechanisms can be transferable; the challenge is to identify which parts of the context are salient to mechanisms so that they may be replicated in other settings. This is why we adapted our methods to articulate specific strategies that appear to trigger mechanisms in each context. For example, we found in Kuopio, Finland, that having a city mandate that endorsed collaboration across organizations was helpful in facilitating HiAP implementation since it conveyed authority and credibility for those aims.^13^ While having the tenets for HiAP included in a city mandate might be more common in places like Finland, another city without a strong history of egalitarianism and without HiAP experience may benefit from aiming to have HiAP (or intersectoral action, more generally) recognized as a solution in local policy documents^14^ to stimulate the same sense of authority and credibility to relevant actors.

 Amri and Bump^3^ suggested that we should have included health equity improvement as an outcome in our analysis. Given that health equity is central to the concept of HiAP, we strongly agree that while important it is rarely made explicit in research and practice. That said, our analysis did capture the broader culture of equity in Finnish governance systems, as exemplified by a comment from a city planner: “We try to put different kind of houses to the area like the rent house to the so-called elite area and like mix up the people because there is lots of survey data that when you put people mixed together in this different social classes, the classes will almost disappear, the kids played together.” Participants in our study did not frequently use the term health equity during interviews (or even HiAP), which highlights the need to learn how to study the nuances of health equity in practice and parlance.

 Greater attention should be paid to understanding how and under what circumstances monitoring and evaluation of health equity processes and outcomes can be integrated into HiAP initiatives, and how to navigate threats to equity, such as changes in political leadership. One challenge for researchers is that often health equity improvement is implicit in the mandate for HiAP (eg, “improving quality of life for everyone”) and health equity (and even HiAP) may not be a familiar concept to local actors, so researchers have to study equity indirectly. Practitioners and researchers alike should acknowledge that the concept of equity is normative, so people and organizations involved in HiAP initiatives may have a hard time speaking the same language when it comes to health equity planning; this can water down or create conflicting approaches to addressing equity.^15^ Finally, health equity outcomes often require a longer period to manifest following upstream interventions, which makes evaluation complicated. Describing theories of change can help researchers and practitioners alike to identify expected short-, medium-, and long-term outcomes so that developmental evaluation approaches can be applied. Also, a systems theory approach could help anticipate which determinants of health are likely to drive those outcomes.

 We cordially thank the authors of the commentaries for their contribution to the growing discourse on HiAP implementation. In response to their comments, we have laid out a research agenda that includes the continued and expanding use of systems theory and realist ontology to capture the complexity of HiAP implementation and wide array of actors involved, and the increased attention to promoting health equity in HiAP initiatives.

## Ethical issues

 Ethics approval was granted from the University of Toronto Research Ethics Board for the original research on which this correspondence is based.

## Competing interests

 Authors declare that they have no competing interests.

## Funding

 The original research on which this correspondence is based was supported by funds from the University of Toronto. Ahmed M. Bayoumi was supported by the Baxter and Alma Ricard Chair in Inner City Health at Unity Health Toronto and the University of Toronto.
